# Evaluation and Management of Thyroid Nodules: A Joint Consensus Statement From the British Thyroid Association (BTA), British Association of Endocrine and Thyroid Surgeons (BAETS) and Collaborating Bodies

**DOI:** 10.1111/cen.70116

**Published:** 2026-03-05

**Authors:** Ram Moorthy, Saba P. Balasubramanian, Kate Farnell, Mairead Kelly, Gitta Madani, Mufaddal Moonim, Carla Moran, Julia Priestley, Michael Stechman, Emma Watts, Kristien Boelaert

**Affiliations:** ^1^ Department of Otolaryngology‐Head and Neck Surgery Wexham Park Hospital, Frimley Health NHS Foundation Trust Slough UK; ^2^ Sheffield Teaching Hospitals NHS Foundation Trust Sheffield UK; ^3^ University of Sheffield Sheffield UK; ^4^ Butterfly Thyroid Cancer Trust NCCC Freeman Hospital Newcastle UK; ^5^ Imaging Department Imperial College Healthcare NHS Trust London UK; ^6^ Department of Histopathology Charing Cross Hospital, Imperial College Healthcare NHS Trust London UK; ^7^ Endocrinology Section Beacon Hospital Dublin Ireland; ^8^ Department of Endocrinology and Diabetes Mellitus St. Vincent's University Hospital Dublin Ireland; ^9^ School of Medicine University College Dublin Ireland; ^10^ British Thyroid Foundation Harrogate UK; ^11^ Department of Endocrine Surgery University Hospital Wales, Health Park Cardiff UK; ^12^ Department of Applied Health University of Birmingham Birmingham UK; ^13^ Department of Otolaryngology‐Head and Neck Surgery Queen Elizabeth Hospital Birmingham, University Hospitals Birmingham NHS Foundation Trust Birmingham UK; ^14^ Department of Endocrinology Queen Elizabeth Hospital Birmingham, University Hospitals Birmingham NHS Foundation Trust Birmingham UK

## 1 Introduction

Thyroid nodules are detectable in up to 68% of the population, with improved access to ultrasonography and technological advancements leading to a surge in the identification of incidental nodules [1, 2, 3]. Reassuringly, fewer than 10% of thyroid nodules are malignant, and the majority of these are indolent microcarcinomas with favourable prognoses that rarely impact overall survival [4, 5].

The 2014 British Thyroid Association (BTA) and 2015 American Thyroid Association (ATA) guidelines offer comprehensive recommendations for the management of thyroid nodules [6, 7]. However, additional data have emerged over the past decade, prompting evaluation of these protocols, as reflected in the updated 2025 ATA guidelines [8, 9, 10, 11, 12, 13, 14, 15, 16]. Both guidelines advocate for the use of ultrasonography to determine whether a thyroid nodule warrants Fine Needle Aspiration Cytology (FNAC). The BTA relies on the ‘U’ grading system based on ultrasound features, without considering nodule size as a criterion for FNAC. In contrast, the ATA incorporates both sonographic patterns and relies on nodule size in determining the need for biopsy. The National Institute for Health and Care Excellence (NICE) has also issued UK‐specific guidance recommending urgent referral via cancer pathways for unexplained thyroid lumps (NG12) and offers detailed protocols for the clinical evaluation of thyroid disease and standardised ultrasound reporting criteria (NG230; NG145) [17, 18].

Incorporating these guidelines into clinical practice may improve thyroid nodule management by minimising overtreatment and avoiding unnecessary invasive procedures including FNAC, core biopsy and surgery. However, despite the availability of comprehensive guidance, significant clinical uncertainty persists. Challenging scenarios include cases in which ultrasound demonstrates indeterminate, suspicious or malignant features (U3−U5), yet cytology yields benign results (Thy2) [5, 19]. The management of sub‐centimetre nodules exhibiting suspicious sonographic features remains nuanced given the limitations of current risk stratification systems and biopsy thresholds [20, 21]. Finally, toxic thyroid nodules may yield indeterminate cytology (Thy3a or Thy3f) due to their hypercellularity and follicular architecture despite their typically low malignancy risk, potentially resulting in overdiagnosis and unnecessary surgery [22, 23].

These persistent areas of clinical uncertainty continue to cause variation in treatment, leading to inconsistent decision‐making, unnecessary interventions and increased patient anxiety. Recognising these challenges, a multidisciplinary working group involving clinicians and patient representatives was convened.

### 1.1 Aim

This consensus statement aims to supplement existing guidelines by providing practical recommendations to address key areas of controversy and promote greater clarity in clinical practice. This consensus highlights 10 key recommendations for practice.

### 1.2 Working Group Members and Organisations

Members of the Working Group, along with their affiliated organisations are listed below.

**Table:** 

Mr Ram Moorthy (Chair) Professor Saba Balasubramanian Mr Michael Stechman Ms Mairead Kelly Ms Emma Watts	British Association of Endocrine and Thyroid Surgeons (BAETS)
Professor Kristien BoelaertProfessor Carla MoranMs Emma Watts	British Thyroid Association (BTA)
Professor Kristien Boelaert	Society for Endocrinology (SfE)
Dr Gitta Madani	British Society of Head and Neck Imaging (BSHNI)
Dr Mufaddal Moonim	UK Endocrine Pathology Society (UKEPS)
Ms Julia Priestley	British Thyroid Foundation (BTF)
Mrs Kate Farnell	Butterfly Thyroid Cancer Trust

## 2 Indications for Thyroid Ultrasound

In alignment with NICE Guideline NG145, thyroid ultrasound should be offered when there is clinical suspicion of malignancy in patients with normal thyroid function and any of the following criteria [18]:

- Thyroid enlargement [6, 16, 24].
- Focal thyroid nodularity detected on clinical examination [16, 24, 25].
- Incidental thyroid abnormalities identified on other imaging modalities and triaged using Duke's 3‐tiered system [6, 26, 27, 28].

Ultrasound evaluation is not routinely indicated for thyroid dysfunction or non‐specific symptoms in the absence of a palpable thyroid mass [29]. However, it may be appropriate in the presence of a palpable thyroid nodule or enlarging neck mass, particularly for patients with thyroid dysfunction where co‐existing structural pathology is suspected (such as lymphoma in Hashimoto's disease) [30, 31].

### 2.1 Role of Thyroid Stimulating Hormone (TSH) in Pre‐Ultrasound Assessment

All patients should have serum TSH measured prior to requesting a thyroid ultrasound, and the result should be included on the request form where available [6]. TSH helps determine thyroid functional status, guiding the need for scintigraphy in hyperthyroid patients and supporting cytological interpretation, particularly where thyrotoxicosis may affect cellular morphology [22, 23, 32, 33]. Further detail is provided in Section 3.

### 2.2 Additional Clinical Indications for Thyroid Ultrasound

Thyroid ultrasound is also indicated in additional scenarios beyond suspected malignancy in euthyroid patients. The following scenarios represent important indications for targeted thyroid ultrasound:

- Suspected progression or morphological change in a known thyroid nodule [16, 18, 24].
- Cervical masses of uncertain origin, including enlarged cervical lymph nodes [6, 16, 24, 34, 35].
- Patients with a proven genetic predisposition or strong family history of thyroid cancer [6, 24, 36, 37].
  ◦ Including PTEN hamartoma tumour (Cowden's) syndrome [38], familial adenomatous polyposis coli (FAP) [39], DICER1 syndrome [40, 41] or Multiple Endocrine Neoplasia (MEN) syndromes due to RET mutations [42].
  ◦ While the role of ultrasound in this population remains uncertain, it may offer value in select cases [16, 43].

- Incidental thyroid nodules detected on CT, MRI or PET CT [6, 26, 27, 28]:
  ◦ For incidental thyroid nodules identified on non‐ultrasound imaging, triage should be undertaken using the Duke 3‐tiered system, below, prior to referral for dedicated thyroid ultrasound [28, 44].
  ◦ Duke 3‐Tiered System:
    ▪ Category 1: Patients should be referred for ultrasound regardless of size if the nodule is FDG‐avid on PET‐CT (see below), demonstrates local invasion, or is associated with suspicious lymphadenopathy.
    ▪ Category 2: For patients < 35 years old, consider referral for ultrasound if the nodule is ≥ 1.0 cm. In paediatric patients, any size nodule warrants further investigation with ultrasound.
    ▪ Category 3: For patients ≥ 35 years old, consider referral for ultrasound if the nodule is ≥ 1.5 cm.
  ◦ Any nodule which does not meet these criteria for ultrasound evaluation should not be mentioned in the conclusion of the original imaging report.

- Focal FDG‐avid thyroid lesions identified on PET‐CT [6, 16, 24, 28, 45].
  ◦ These nodules carry a risk of malignancy of up to 30.8% [46, 47].
  ◦ Further evaluation is recommended with ultrasound and FNAC (if indicated), unless the patient's life expectancy is less than 5 years due to index cancer, age or comorbidities which would preclude further investigation [6, 45].
  ◦ The decision to perform FNAC should be guided by ultrasound risk stratification, rather than positron emission tomography (PET) uptake alone [45].

## 3 Standardisation of Thyroid Ultrasound Reporting

Thyroid ultrasound examinations should be conducted and reported in a standardised manner to ensure consistent, high‐quality assessments to optimise clinical decision‐making [48, 49]. All ultrasound reports should include information relevant to diagnosis, risk stratification and ongoing management. Where applicable, comparisons with prior imaging should be documented, with specific reference to changes in nodule size, morphology or other sonographic features [50, 51].

The following elements are considered essential and should be included in all thyroid ultrasound reports [18]:

- Risk stratification system: The report must clearly state and use a recognised thyroid nodule risk stratification system [52, 53]. This system should be selected in alignment with the local thyroid multidisciplinary team (MDT) to promote consistency in assessment and minimise the risk of unwarranted FNAC [53, 54]. Examples include the BTA U score [6], ACR TI‐RADS [8] or EU‐TIRADS [9]. A single, consistent system should be used across all operators reporting to the MDT [55, 56].
- Nodule characterisation: Each nodule assessed should have its size, grade and sonographic features which determine the assigned score, clearly documented [6, 16, 24, 57]. In the context of multinodular goitre, focus should be placed on nodules with suspicious characteristics [16, 58].
- Nodule location: The anatomical location of any nodule(s) that has undergone or is scheduled for FNAC must be clearly described [6, 16, 24].
- Bilateral assessment: Both thyroid lobes should be evaluated and described in the report, including documentation of normal findings if present [59, 60].
- Cervical lymphadenopathy risk assessment: An overall assessment of malignancy risk in the neck should be provided. This must include detailed documentation of any abnormal lymph nodes, specifying their location within the central compartment (Levels VI and VII) or lateral neck (Levels I−V) and sonographic characteristics [6, 16, 24, 59]. If lymph nodes appear normal, this should be explicitly stated [35].
- Vascularity: Doppler vascularity is a poor standalone predictor of malignancy, and its inclusion may lead to misclassification and unnecessary FNAs [61]. While vascular assessment may occasionally provide supplementary information in challenging cases, we recommend that vascularity is not used as a primary feature in nodule risk stratification, in line with other international risk stratification systems [8, 9].

## 4 Indications for FNAC

A clearly defined protocol should be established to determine which thyroid nodules warrant FNAC. This should be based on the risk stratification system agreed upon by the local Thyroid MDT. The following recommendations apply primarily to institutions using the BTA 2014 guidelines for ultrasound grading [6]. According to these guidelines, FNAC is indicated for all nodules graded U3 (indeterminate), U4 (suspicious) or U5 (malignant). Additional FNAC may be considered for benign (U2) nodules if clinical features suggest an increased risk of malignancy, such as ipsilateral vocal cord palsy or suspicious cervical lymphadenopathy.

### 4.1 Nodule Size

International risk stratifications systems including ACR TI‐RADS [8], EU TI‐RADS [9] and the ATA guidelines [16] incorporate nodule size thresholds when determining the need for FNAC. Aspiration is typically recommended for nodules ≥ 10−25 mm depending on the ultrasound‐based level of suspicion [8, 9, 16, 62, 63]. Nodules < 10 mm are not routinely biopsied, despite limited evidence suggesting nodule size correlates with risk of malignancy [64, 65, 66, 67, 68, 69]. Although the BTA does not specify size thresholds, it is recognised that small nodules may be safely monitored in select cases [16, 70]. Therefore, the following consensus recommendations are offered to balance diagnostic efficacy, patient safety and resource availability:

- U3 Nodules < 1 cm: May be discharged if asymptomatic and without suspicious clinical features. Follow‐up ultrasound should be reserved for cases with significant clinical suspicion [5, 6, 19].
- U3 Nodules 1−2 cm: May undergo either FNAC or follow‐up ultrasound, according to local protocols [6, 16, 71].
- U3 Nodules > 2 cm: Should be considered for FNAC [6, 9, 16, 72].
- U4/U5 Nodules 5−10 mm: May be considered for FNAC or follow‐up ultrasound based on local MDT policy and concerning clinical or sonographic features [72, 73].
- U4/U5 Nodules > 1 cm: Should be considered for FNAC [6, 9, 16, 72].

### 4.2 Cystic Nodules

In cystic nodules, particularly those classified as benign on ultrasound, any aspirated fluid should be sent for cytological analysis rather than discarded [74, 75]. Aspiration, thermal or ethanol ablation may be offered for symptomatic relief [18, 76, 77]. However, confirmation of benign cytology is required prior to ablation [78].

### 4.3 FNAC Technique and Quality Assurance

The technique for performing FNAC may vary between institutions. However, routine audit of FNAC outcomes is strongly recommended, with a particular focus on reducing non‐diagnostic (Thy1) rates [75, 79]. Quality metrics should include the proportion of diagnostic (Thy2−Thy5) versus non‐diagnostic (Thy1) results [80, 81].

Thyroid nodule assessment clinics should aim for a minimum FNAC adequacy rate of 85% in line with national benchmarks [81, 82, 83]. This should be monitored and audited regularly as part of ongoing quality assurance [79].

### 4.4 Limitations of FNAC in Non‐Papillary Thyroid Neoplasms

FNAC is highly effective for diagnosing papillary thyroid carcinoma, but has limited sensitivity for non‐papillary lesions, in particular the discrimination of follicular thyroid carcinoma from follicular adenomas and hyperplastic nodules [75, 84]. False‐negative rates for malignancy vary by cytological category [84, 85]:

- Thy1: 5%−12%
- Thy2: 0%−5%
- Thy3a: 25%
- Thy3f: 31%

These limitations highlight the importance of clinical and radiological correlation in the interpretation of FNAC results, especially in indeterminate cases.

### 4.5 Toxic (Hyperfunctioning) Nodules

Due to the follicular nature of autonomous (toxic, ‘hot’) thyroid nodules, it is not unusual to obtain a cytological report of indeterminate malignancy risk (Thy3a/Thy3f) [22, 23, 32, 33]. Confirmed autonomous toxic thyroid nodules are rarely malignant and should therefore not routinely undergo FNAC, in contrast to similar nodules in euthyroid patients [22, 23, 86]. Clinical decision‐making should incorporate thyroid function tests and radionuclide scintigraphy findings to guide appropriate care and avoid unnecessary intervention [16]. In this context, FNAC, repeat FNAC or surgical referral should be approached with caution, and only considered when there are additional clinical or radiological features of concern [23, 72].

## 5 Indications for Core Biopsy

FNAC remains the investigation of choice for the initial pathological evaluation of thyroid nodules; this well‐established, minimally invasive technique is highly effective in triaging nodules for further management [18, 87, 88]. However, core needle biopsy may be appropriate in specific clinical scenarios where FNAC is insufficient or non‐diagnostic [89, 90, 91]. Core biopsy should be considered in the following situations:

- Suspected locally aggressive thyroid malignancy: Core biopsy is indicated for rapidly enlarging nodules or suspected extra‐thyroidal extension when FNAC has failed to establish a diagnosis or additional tissue is required for molecular or immunohistochemical staining [6, 89, 92, 93, 94].
- Suspected thyroid lymphoma: Core biopsy may be indicated when the thyroid is the sole or most accessible site for diagnostic confirmation [6, 95, 96].
- Evaluation of fibrosing thyroid disorders: Including Riedel's thyroiditis or other fibrosing processes, where FNAC has proven inconclusive [97, 98].
- Repeated non‐diagnostic FNAC results: Where two or more FNACs are reported as non‐diagnostic (Thy1) in a nodule classified as suspicious (U4) or malignant (U5), core biopsy may be considered following MDT discussion and agreement [50, 90, 99, 100].

For the assessment of fibrosing lesions and lymphoma, 16‐gauge core needles are generally considered optimal for tissue adequacy [101]. Caution is advised when performing core biopsies on follicular‐patterned lesions as interpretation relies on histological evaluation of the lesional capsule for evidence of invasion [102]. Needle disruption of the capsule may create artefacts that mimic capsular or vascular invasion, potentially leading to diagnostic error [103, 104, 105]. Pathologists should be made aware of this potential pitfall to avoid overinterpretation [106].

Core needle biopsy is generally considered a safe procedure [107]. While the risk of tumour seeding is slightly higher with core biopsy (0.0011%) compared to fine needle aspiration (0.00012%), it remains extremely low overall [108]. FNA should remain the first line technique, and consideration of core biopsy should be guided by diagnostic necessity, the clinical context and the potential added value of core tissue sampling. All histopathology reports from thyroid core biopsies should be categorised using the most recent Royal College of Pathologists guidance to ensure consistency and clinical utility [75, 105, 109].

## 6 Thyroid Nodule Assessment Pathway

All patients referred to secondary care with a suspected thyroid nodule should ideally be assessed in a consultant‐led thyroid nodule assessment clinic [110]. At the initial visit:

- Patients should be evaluated by a thyroid surgeon or endocrinologist with expertise in thyroid disease [111].
- Patients should undergo ultrasound assessment with FNAC performed where appropriate by a practitioner suitably trained in thyroid imaging and intervention [112, 113].
- All clinicians involved should be core members of the thyroid MDT [6].

## 7 Composition of the Thyroid MDT

The following specialists should constitute the core membership of the Thyroid MDT and be present for all meetings [114]:

- Thyroid surgeon(s)
- Endocrinologist
- Oncologist
- Pathologist (covering both cytopathology and histopathology)
- Radiologist
- Clinical nurse specialist (CNS)
- MDT co‐ordinator

Additional MDT members may be invited as required based on specific patient circumstances. This may include, but is not limited to, a paediatric endocrinologist, a paediatric oncologist and a PET or nuclear medicine specialist [115]. Where cytopathology and histopathology are reported by different consultants, representation from both specialities should be present at the MDT [75].

## 8 Indications for MDT Review

Whilst practice may vary across centres, the following principles are recommended to support consistent, effective and efficient MDT discussion without compromising individualised care.

### 8.1 Cases for Mandatory MDT Discussion

The following cases should be discussed at the Thyroid MDT:

- All patients with histologically confirmed thyroid malignancy [116].
- Patients with cytological classification of Thy4 or Thy5 [75].
- Cases demonstrating discordance between clinical presentation, ultrasound findings and cytology [6].
- Nodules with Thy3a or Thy3f cytology where proposed management deviates from established local or national protocols [117].

### 8.2 Minimum Information for MDT Discussion

To ensure efficient and informed case review, the following information should be available for all patients discussed at the Thyroid MDT:

- TSH [118].
- Ultrasound report (must include assessment of the contralateral lobe and status of the lateral and central neck lymph nodes) [6].
- FNAC and/or histology [75].
- Cross‐sectional imaging (if ultrasonographic suspicion of nodal or distant metastases or when thyroid cancer is staged as T3 or T4) [6, 117].

Providing complete clinical and imaging information helps prevent delays and minimises the need for repeated discussion across multiple meetings.

### 8.3 MDT Protocols to Support Decision Making

MDTs should develop and implement clear protocols and decision‐making pathways to support timely and effective case management [116]. These should be designed to streamline the discussion of routine cases while ensuring adequate time is allocated for more complex presentations. All decisions must be formally documented and subject to regular audit to support quality assurance and service improvement [116].

Where local protocols deviate from national guidance, MDTs should clearly define their approach based on local audit data, available expertise and institutional outcomes. For example, recent evidence suggests the risk of malignancy for Thy3a and Thy3f nodules may differ from historical estimates, highlighting the importance of reviewing local diagnostic outcomes [119, 120, 121]. Given that the BTA guidelines recommend repeat FNAC for Thy3a nodules and diagnostic surgery for Thy3f, any deviation from these protocols should be discussed at the MDT [6].

## 9 FNAC and Histopathology Review in the MDT Setting

To ensure consistency and diagnostic accuracy, the following review processes are recommended:

- FNAC with Thy4 or Thy5 cytology: Must be reviewed by a pathologist who is a core member of the thyroid MDT [6, 75].
- FNAC with Thy3a or Thy3f cytology: Review by a core pathology MDT member is recommended as good practice [75]. However, if samples are not reviewed individually, a standardised MDT protocol must be in place to guide management (as described above). Any deviation from this protocol should prompt a formal MDT discussion.
- Histology from diagnostic hemithyroidectomy: Must be reviewed by a pathologist who is a core member of the thyroid MDT [117].
- Benign cytology and histology results: Cytology like Thy2 or benign histology does not routinely require MDT discussion unless there is ongoing clinical concern, the case has been flagged for MDT review previously, or a significant discrepancy exists between imaging and cytology (including Thy2 nodules with U4/U5 sonographic features) [85, 122, 123, 124].

### 9.1 Quality Assurance

The rate of non‐diagnostic FNACs (Thy1, excluding Thy1c) should be monitored regularly. The proportion of Thy1 reports should not exceed 15% of all thyroid FNACs [75]. If rates are consistently above this threshold, services should consider implementing Rapid On‐Site Evaluation (ROSE) as a quality improvement measure [81, 83, 117, 125].

## 10 Recommended Frequency of MDT Discussion

It is recommended that MDT meetings are held at least every 1−2 weeks to ensure timely discussion and appropriate patient management.

## 11 Surveillance Strategy for Conservatively Managed Thyroid Nodules

There is limited evidence defining the optimal frequency, interval or duration of ultrasound surveillance for patients with thyroid nodules managed without surgery [126]. This contributes to considerable variation in clinical practice across international settings [6, 16, 24, 127, 128]. In the United Kingdom, thyroid nodules are typically not subject to routine follow‐up or repeat FNAC unless there is significant clinical concern or suspicious ultrasound characteristics suggestive of malignancy [6]. By contrast, the ATA advocates repeat ultrasound at 12−24 months for nodules demonstrating even low to intermediate suspicion on ultrasound [16].

Recognising this, the consensus group offers the following recommendations, acknowledging that local resource limitations, clinician experience and patient factors will influence practice. For example, some clinicians may advocate for FNAC to facilitate earlier discharge, while others may favour continued ultrasound surveillance [19, 129, 130].

### 11.1 General Recommendations

Patients with thyroid nodules should be risk‐stratified based on clinical presentation and radiological or cytological features. The following guidance outlines recommended follow‐up strategies for nodules deemed benign or low‐risk:

- Patients with benign (U2) thyroid nodules which do not cause compressive symptoms and do not require thermal ablation or surgery may be discharged without further follow‐up [6, 131, 132].
- If follow‐up is recommended for benign or low‐risk nodules, a repeat ultrasound should be performed at 12 months [6, 16, 131]. Earlier scanning is not considered beneficial, given that most benign and malignant nodules remain stable during conservative management [133, 134, 135].
- The frequency and duration of follow‐up should be tailored based on [6, 16, 24, 130, 136]:
  ◦ Ultrasonographic and/or cytological features of the nodule.
  ◦ Development of new symptoms or lymphadenopathy.
  ◦ Underlying clinical risk factors.
  ◦ Evidence of progression or growth on follow‐up imaging.
  ◦ The underlying health status of the patient and comorbidities.

### 11.2 Suggested Follow‐Up Strategies by Nodule Type

The following stratified approach offers practical guidance for surveillance intervals and discharge decisions across different nodule types, with an emphasis on safety and avoidance of unnecessary intervention:

- U2 nodule > 1 cm with high‐risk clinical risk factors: Consideration of further follow‐up and/or FNA should be made on an individual basis, depending on clinical risk factors. High‐risk clinical features include, but are not limited to, a strong family history of thyroid cancer (i.e., two or more first‐degree relatives affected, or thyroid cancer diagnosed in one first‐degree relative during childhood or adolescence), a history of childhood or young‐adult head and neck irradiation and an isolated nodule in men < 35 years old [5, 6, 16, 137, 138, 139].
- U3 nodule > 1 cm with Thy2 cytology results: The malignancy risk for U3 nodules is up to 22%, but when combined with Thy2 cytology, the risk is reduced to less than 10% [5, 19]. Repeat FNAC is not mandated when a single Thy2 result is supported by two stable ultrasounds or the patient has two consecutive Thy2 results [117, 140]. Discharge is appropriate if the nodule is stable on repeat ultrasound at 12 months [122, 132, 141, 142, 143]. Repeat FNAC is, however, recommended in the presence of ipsilateral lymphadenopathy, vocal cord palsy, interval growth or change in sonographic appearance [6, 16, 24]. Local protocols should guide the need for repeat FNAC or follow‐up imaging [122, 143].
- U3 nodule > 1 cm with two consecutive Thy1 cytology results: Recommend at least 2 years of ultrasound follow‐up or consider diagnostic hemithyroidectomy following shared decision‐making [19, 117, 143].
- U3 nodule > 1 cm without cytology (FNAC not indicated or performed): Consider at least 2 years of ultrasound follow‐up before discharge [5, 19].
- U4 or U5 nodule < 1 cm without cytology (FNAC not indicated or performed): Recommend at least 2 years of ultrasound follow‐up before considering discharge [19, 144, 145].
- Thy3a, Thy3f, Thy4 or Thy5 cytology managed conservatively: Most of these nodules will undergo diagnostic or therapeutic surgery. If surgery is not performed, a minimum of 5 years of follow‐up is recommended, with annual ultrasound surveillance [19, 146, 147].

## 12 Conclusion

Despite the availability of national and international guidance, significant variation persists in the evaluation and management of thyroid nodules. This is driven by limitations within existing risk stratification systems, interpretive challenges related to cytology and uncertainty regarding optimal surveillance strategies for nodules managed non‐surgically.

This multidisciplinary consensus statement provides a series of practical, evidence‐informed recommendations to address key areas of clinical ambiguity. It aims to support the standardisation of care, reduce unnecessary interventions and improve patient experience and outcomes. By integrating current guidelines with expert consensus and emerging evidence, this statement serves as a framework to guide consistent and effective thyroid nodule management across the UK. Ongoing audit, MDT collaboration and clinician‐patient shared decision‐making remain central to delivering high‐quality, individualised care.

## Funding

The authors received no specific funding for this work.

## Ethics Statement

The authors have nothing to report.

## Consent

The authors have nothing to report.

## Conflicts of Interest

The authors declare no conflicts of interest.

## Supporting information

Investigating_thyroid_nodules_PIL_v5_final.docx.

## Data Availability

Data sharing is not applicable to this article as no data sets were generated or analysed during the current study.
