# Supplementary material for: Evaluation and Management of Thyroid Nodules: A Joint Consensus Statement From the British Thyroid Association (BTA), British Association of Endocrine and Thyroid Surgeons (BAETS) and Collaborating Bodies
Source: Clin Endocrinol (Oxf). 2026 Mar 5;104(6):682–92. doi: 10.1111/cen.70116 (PMC13124684; doi:10.1111/cen.70116)
Supplement: Supplementary file 1 — Investigating_thyroid_nodules_PIL_v5_final.docx. [file CEN-104-682-s001.docx]

**Consensus statement: Investigating thyroid nodules**

**Information for patients**

The thyroid gland is a small butterfly shaped gland which is found in the front of your neck, just below your Adam’s apple. Thyroid nodules, or lumps, are common, and more than 9 in 10 are found to be benign (not cancer). The main cause of thyroid nodules is unknown, but they are more common in women than in men and occur more frequently as people get older.

Some thyroid nodules are noticeable and can be seen or felt in the neck, but many are only picked up during examinations or scans for other investigations . Although the majority are not cancer it is important that all nodules on your thyroid are investigated by a specialist doctor without delay.

This leaflet aims to give you:

- an overview of the tests you can expect as part of an investigation of thyroid nodules
- information about what will happen following the tests
- what follow up care you should expect

**What tests will I have?**

At your hospital appointment you will have a consultation with a doctor who will ask you about your symptoms and examine your neck. As part of the investigation of the nodule you can also expect to have a number of tests. These tests may be carried out over a number of different appointments but in some hospitals there may be a clinic which is able to perform all or most of the investigations in one visit. This is known as a ‘One-Stop Clinic.’

Your doctor will explain to you which tests you will have and why they are being carried out. They will also tell you when you can expect to receive the results and how you will receive them. If you have any questions about any of this your doctor will be able to answer them.

*You should be given contact details (telephone or email) so that you can get in touch with your doctor’s secretary if you have any questions.*

Name of contact:

Telephone:

Email:

Thyroid nodules are investigated with some or all of the following tests:

**Thyroid blood test** – this is usually the first investigation you will have and it will measure the level of thyroid hormones in your blood. It does not test for cancer.

**Ultrasound scan (USS)** – this is a painless procedure in which a small probe passes over your neck to examine the structure of your thyroid gland. It can establish the number and size of nodules in the thyroid and can give important information on the likelihood that a nodule is benign or cancerous. The USS will be performed by a specialist in USS (sonographer) or specialist doctor and a formal report will be produced.

**Fine needle aspiration (FNA**) – this is where some cells are removed from a thyroid nodule using a fine needle. The cells are then examined under a microscope. This type of procedure is also known as a biopsy. Local anaesthetic is often given, but you may still feel some discomfort or pressure on your neck during the procedure. Not all nodules need an FNA. If your doctor decides that you do not need an FNA this will be discussed with you. Sometimes the results of the FNA are not conclusive and the test may need to be repeated.

**Core biopsy** – sometimes the sample of cells collected in the FNA biopsy does not provide enough information to assess the nodule properly. In this case you may be asked to have a core biopsy which will collect a bigger sample. A core biopsy is considered a safe procedure and it rarely causes complications. You will probably have a local anaesthetic to numb the area and should not feel any pain but may feel some discomfort while it is being performed.

**What you need to know after your biopsy**

After the biopsy you may feel some discomfort in your neck, especially when you swallow. This will improve quickly and after 48 hours any symptoms should have gone. Simple painkillers such as paracetamol may be helpful. You may also have some bruising around the area where the needle was inserted but this will clear within about a week.

If the lump in your thyroid was mainly caused by fluid, this may be removed (aspirated). It is not unusual for the fluid to collect again over a period of one or more weeks. This should not alarm you, unless it is painful and red, in which case you should contact your GP for advice.

**When will I find out the results of the tests?**

After the necessary tests have been performed, specialist doctors will prepare reports about the results and these will be sent to your doctor. This can take up to three weeks. Your doctor will discuss the information with you at your next appointment, or if you have asked for it to be sent to you by letter or telephone, as soon as the result becomes available. If you have not been notified after three weeks, please contact the consultant’s/doctor’s secretary.

**What are the possible outcomes of the investigations?**

In many cases the biopsy results will indicate the thyroid nodule is benign (not cancer). Sometimes the results are unhelpful, either because there were not enough cells collected or it was not possible to decide on the likelihood of cancer in the nodule based on the cells collected. If this happens your doctor may recommend repeating the biopsy or you may be advised to have an operation to remove part of your thyroid; this is called a lobectomy or hemithyroidectomy. Occasionally, the biopsy will show that the thyroid nodule contains some malignant (cancerous) cells, however not all thyroid cancers can be diagnosed by an FNA test.

**What happens if the biopsy result is suspicious or shows cancer?**

Your doctor will discuss this with you and explain that in most cases the treatment will be the removal of part or all of your thyroid gland (either a hemi-thyroidectomy or a total thyroidectomy). If you would like further information about thyroid cancer please see the resources provided by the patient organisations listed at the end of this leaflet.

**What happens if the biopsy result is benign (not-cancer)?**

If the results confirm that your thyroid nodule is benign your doctor will usually discharge you from their thyroid clinic. You should be very reassured that your nodule is benign as it means there is no evidence of thyroid cancer. The risk of you developing thyroid cancer in the future is very small and no different from anybody else.

**What happens if my doctor decides to monitor my nodule?**

In cases where surgery is not required, your doctor may decide that it is best to monitor your nodule. This is usually done so that the doctor can obtain further reassurance that the nodule is benign. Typically, this will be with a follow up USS. In these cases your doctor will discuss the reasons for monitoring the nodule with you, and how and when the monitoring will be performed.

**Do I need to look out for anything in the future?**

Sometimes thyroid nodules do return and if you become aware of any changes to your neck area you should not ignore them. Symptoms to look out for include:

- the nodule is getting bigger
- the nodule feels harder
- a new lump appears
- you develop difficulties swallowing or breathing
- your voice becomes hoarse

If you have any of these symptoms, you should make an appointment with your GP who will examine your neck and advise whether you should be reviewed by a specialist again for further tests.

**Further information**

Evaluation and Management of Thyroid Nodules: A Joint Consensus Statement from the British Thyroid Association, British Association of Endocrine and Thyroid Surgeons and Collaborating Bodies *[link to document]*

British Thyroid Foundation’s Guide to Thyroid Nodules and Swellings [*insert hyperlink* [*https://www.btf-thyroid.org/thyroid-nodules-and-swellingsleaflet*](https://www.btf-thyroid.org/thyroid-nodules-and-swellingsleaflet) ]

**Patient support**

The following patient organisations provide information and peer support:

*British Thyroid Foundation*<https://www.btf-thyroid.org/>

*Butterfly Thyroid Cancer Trust*<http://www.butterfly.org.uk/>

*Thyroid Cancer Support Group – Wales*[*https://thyroidsupportwales.co.uk/*](https://thyroidsupportwales.co.uk/)

*Association for Multiple Endocrine Neoplasia Disorders (AMEND)*<https://www.amend.org.uk/>**Glossary**

Biopsy – a sample of tissues or cells that can be examined underneath a microscope.

Core biopsy – a biopsy in which a cylindrical sample or tissue or cells is obtained by a hollow needle.

Fine needle aspiration (FNA) – this is a type of biopsy where cell samples are taken through a very thin needle. Sometimes this is termed FNAC (fine needle aspiration cytology)

Hemithyroidectomy (also called a lobectomy) - this is the surgical removal of part of the thyroid. This is sometimes necessary when other tests are inconclusive regarding the likelihood of cancer within the nodule

Total thyroidectomy – this is the removal of the whole of the thyroid gland by surgery

Thyroid blood tests – blood tests to measure the level of thyroid hormones in the blood, also called thyroid function tests (TFTs)

Ultrasound scan (USS) - a scan with a small probe that passes over your neck to assess the thyroid gland and lymph nodes. It sends sound waves into the neck tissue which are transferred into an image that shows the different structures in the neck. It does not involve radiation exposure.
